# A Simple and Robust MRI Radiomics Feature for Predicting Pathological Complete Response: A Proof‐of‐Concept Study in Breast Cancer Neoadjuvant Chemotherapy

**DOI:** 10.1002/cnr2.70562

**Published:** 2026-05-22

**Authors:** Hongen Li, Li Zhang, Yihui Zeng, Yuanyuan Chen, Xiaosong Jiang, Ruoxian Zhang, Yan Zhang

**Affiliations:** ^1^ Department of Radiology Guangdong Women and Children Hospital/Women and Children's Hospital, Southern University of Science and Technology Guangzhou China

**Keywords:** breast cancer, complete pathological remission, magnetic resonance imaging, neoadjuvant chemotherapy, radiomics

## Abstract

**Background:**

Early prediction of pathological complete response (pCR) to neoadjuvant chemotherapy (NAC) in breast cancer remains challenging. This study aimed to explore the value of a radiomics model based on dynamic contrast‐enhanced magnetic resonance imaging (DCE‐MRI) acquired after the second cycle of NAC for early prediction of pCR.

**Methods and Results:**

A retrospective analysis was conducted on 119 breast cancer patients who underwent NAC at our hospital between March 2020 and August 2023. Patients were categorized into pCR and non‐pCR groups based on postoperative Miller–Payne pathological grading as the gold standard. Tumor regions of interest (ROIs) were manually delineated on phase‐three DCE‐MRI sequences. PyRadiomics extracted 851 features. A rigorous dimensionality reduction process—including stability screening, intergroup differential analysis, and decorrelation analysis—yielded 88 key features. LASSO regression (10‐fold cross‐validation) ultimately selected three optimal wavelet‐based texture features that formed the core components of our radiomics signature: wavelet. LLH_glcm_Idn (inverse difference normalized), wavelet. LLH_glcm_MCC (maximum correlation coefficient), and wavelet. LHL_firstorder_Skewness. The dataset was randomly split into a training set (83 cases) and a validation set (36 cases) at a 7:3 ratio. A support vector machine (SVM) classifier was constructed, and model performance and clinical utility were evaluated using receiver operating characteristic (ROC) curves and decision curve analysis. Among 119 patients, 43(36.13%) achieved pCR. The constructed radiomics model demonstrated an area under the curve (AUC) of 0.667 and 0.647 in the training and validation sets, respectively, with accuracy rates of 66.27% and 73.49%. Decision curve analysis suggested potential clinial utility under hypothetical scenarios when the probability threshold exceeded 0.3, although this finding is exploratory and requires prospective validation.

**Conclusion:**

This study developed and internally validated a minimalist radiomics model based on mid‐treatment MRI, demonstrating moderate and stable predictive capability for pCR after NAC in breast cancer and showing potential for aiding clinical decision‐making. As an exploratory proof‐of‐concept study, the findings underscore the necessity for future multicenter external validation and integration of multimodal features.

## 1 Introduction

Neoadjuvant chemotherapy (NAC) has become the standard treatment regimen for locally advanced breast cancer (LABC), with one of its primary goals being the achievement of pathological complete response (pCR). Attaining pCR is closely associated with significantly improved disease‐free survival and overall survival, making it a key prognostic indicator [1]. Therefore, accurately predicting pCR outcome early in NAC is crucial for achieving personalized treatment, avoiding the toxic side effects of ineffective chemotherapy in non‐responders, and optimizing surgical decisions (e.g., considering breast‐conserving surgery or clinical trials for surgery exemption) [2]. Currently, the gold standard for pCR assessment is postoperative pathology, but this invasive, retrospective method cannot provide real‐time guidance for dynamic treatment adjustments during therapy. Conventional imaging methods, such as dynamic contrast‐enhanced magnetic resonance imaging(DCE‐MRI), are recommended for monitoring NAC response but primarily rely on morphological and dimensional changes (e.g., RECIST criteria) or semi‐quantitative kinetic curve analysis [3]. These approaches suffer from high subjectivity, limited sensitivity to early microscopic changes, and poor consistency with final pathology. As an emerging quantitative analysis method, radiomics enables high‐throughput extraction of numerous texture, shape, and heterogeneity features from conventional medical images that are imperceptible to the human eye [4]. These features may reflect intrinsic tumor biological characteristics (e.g., cellular density, necrosis, heterogeneity), thereby sensitively capturing early changes in tumor response to chemotherapy [5]. Recent studies have demonstrated the significant potential of radiomics features derived from pretreatment or early‐treatment MRI in predicting pCR after NAC in breast cancer [6, 7]. However, most research has focused on pretreatment baseline scans or posttreatment evaluations. Relatively few studies have explored radiomics analysis at the critical decision point during mid‐NAC treatment (e.g., after the second cycle), despite the importance of timely prediction at this stage for adjusting treatment regimens. Furthermore, existing models exhibit considerable performance variability with limited multicenter validation. Therefore, this study aimed to develop and internally validate a radiomics model based on DCE‐MRI after the second cycle of NAC for early prediction of pCR status in breast cancer patients. Through rigorous feature selection and machine learning processes, we constructed a minimalist predictive model and evaluated its discriminatory efficacy and clinical utility. This exploratory, single‐center retrospective analysis provides preliminary evidence for the feasibility of this predictive strategy and lays the groundwork for future large‐scale prospective validation. As an exploratory proof‐of‐concept study focusing specifically on the mid‐treatment clinical decision window, we deliberately limited our analysis to a single time point (after the second NAC cycle). We acknowledge that validation across multiple time points would provide stronger evidence and will be essential in future studies.

## 2 Materials and Methods

### 2.1 Case Information

This study is a single‐center, retrospective, observational investigation approved by the Ethics Review Committee of Guangdong Maternal and Child Health Hospital (No. [202401244]). Patient informed consent was waived due to the retrospective analysis of anonymized data. This exemption complies with the Declaration of Helsinki and relevant ethical guidelines for retrospective studies in China.

We retrospectively screened consecutive cases of breast cancer patients who underwent NAC and completed surgery at our hospital between March 2020 and August 2023. Inclusion criteria: ① pathologically confirmed primary invasive breast cancer via core needle biopsy; ② received standard NAC regimen at our institution and underwent breast MRI (including DCE sequences) at baseline (before NAC initiation) and within 1 week after the second cycle; ③ underwent radical surgery (mastectomy or breast‐conserving surgery) within 6–12 weeks after NAC completion with a complete postoperative pathology report; and ④ complete clinical and pathological data (including age, molecular subtype, hormone receptor status, HER2 status, Ki‐67 index, etc.). Exclusion criteria: ① poor MRI image quality with severe artifacts affecting lesion delineation; ② failure to complete the planned NAC course (e.g., due to disease progression, severe toxicity, or personal reasons); ③ history of prior treatment for ipsilateral breast cancer (surgery, radiotherapy, etc.); and ④ missing clinical or pathological data. Ultimately, 119 female patients were included in the analysis. Patient ages ranged from 25 to 77 years, with a mean age of (48.62 ± 10.26) years. The 119 patients were randomly divided into a training set (83 cases) and a testing set (36 cases) at a 7:3 ratio using a random seed number set in R software (version 4.0.2).

### 2.2 Inspection Methods

A Philips 3.0 T Ingenia MRI scanner (the Netherlands) equipped with a 16‐channel breast‐specific phased array coil was utilized. The scanning sequences and parameters were as follows: (1) Transverse T1WI: TR 400–600 ms, TE 8 ms, slice thickness 4 mm, interslice gap 0.4 mm, 40 slices, matrix 212 × 240, field of view (FOV) 200 × 320 mm, and 1 excitation; (2) fat‐suppressed transverse T2WI: TR 3000–5000 ms, TE 75 ms, slice thickness 4 mm, interslice gap 0.4 mm, 40 slices, matrix 192 × 221, FOV 190 × 320 mm, and 1 excitation; (3) cross‐sectional diffusion‐weighted imaging (DWI): TR 6550 ms, TE 99 ms, slice thickness 4 mm, interslice gap 0.4 mm, 40 slices, matrix 212 × 142, FOV 400 × 340 mm, 1 excitation, and *b*‐value 0 and 800 s/mm^2^; (4) transverse DCE‐MRI: TR 4.4 ms, TE 2.2 ms, slice thickness 1.6 mm, interslice gap −0.8 mm, 187 slices, matrix 328 × 392, FOV 280 × 339 mm, and 1 excitation. Gadoterate meglumine was administered as the contrast agent at a dose of 0.2 mmol/kg body weight, with an injection rate of 2.0 mL/s, followed by a 15 mL saline flush at the same rate. Dynamic contrast‐enhanced scans were performed across six phases post‐injection.

### 2.3 Image Analysis

Images were transferred from the scanner to the picture archiving and communication system (PACS) workstation and analyzed independently in a double‐blind manner by two associate radiologists, each with over 10 years of experience in breast diagnostics.

### 2.4 Pathological Evaluation

Clinical staging of breast cancer followed the American Joint Committee on Cancer (AJCC) 8th edition guidelines, categorizing cases from Stages I to IV [8]. The efficacy of NAC was assessed according to the Miller–Payne grading criteria for pathological response [9, 10]: Grade 1 indicated no significant change in histomorphology or tumour cell count; Grade 2 indicated a reduction in tumour cells of ≤ 30%; Grade 3 indicated a reduction between 30% and 90%; Grade 4 indicated a ≥ 91% reduction in tumour cell count; and Grade 5 indicated no residual malignant cells or only minimal intraductal carcinoma. Grades 1–4 were classified as non‐pathological complete response (non‐pCR), while Grade 5 indicated a pCR.

### 2.5 Radiomics Analysis

#### 2.5.1 Image Segmentation and Feature Extraction

Using ITK‐SNAP software (version 3.60), a senior physician manually delineated the entire tumor volume as regions of interest (ROIs) layer by layer on the Phase‐3 DCE‐MRI sequences. A second associate chief physician verified the ROIs. When tumor boundaries were inconsistent, a third associate chief physician finalized the boundaries through collaborative discussion based on lesion location and morphology. Subsequently, the PyRadiomics toolbox (version 2.20) was employed to automatically extract 851 radiomics features from each ROI. These included shape features, first‐order statistical features, texture features (gray‐level co‐occurrence matrix [GLCM], gray‐level run matrix [GLRLM], gray‐level size‐region matrix [GLSZM], and neighborhood gray‐level difference matrix [NGTDM]), and features derived from the wavelet transform (including eight decompositions: LLL, LLH, LHL, LHH, etc.).

#### 2.5.2 Feature Preprocessing and Selection

All features underwent *Z*‐score normalization. Feature selection was conducted in two steps to ensure robustness and prevent overfitting: Step 1 (stability screening): first, stable features with near‐zero variance were removed, retaining 760 features. Subsequently, a two‐sample *t*‐test or Mann–Whitney *U* test (based on data normality) was performed to remove features showing no significant difference (*p* ≥ 0.05) between the pCR and non‐pCR groups, retaining 230 features. Step 2 (dimension reduction and final feature selection): Pearson correlation analysis was performed on the remaining features. If the absolute value of the correlation coefficient between any pair of features exceeded 0.9, one feature was randomly removed to eliminate high multicollinearity. After these steps, 88 relatively stable, discriminative, and low‐redundancy key features were retained. Finally, the 88 features underwent further contraction and selection using the least absolute shrinkage and selection operator (LASSO) regression (10‐fold cross‐validation, with the optimal regularization parameter *λ* selected based on minimizing binomial bias). LASSO compresses the coefficients of unimportant features to zero, ultimately yielding a set of nonzero coefficients representing the most predictive features.

#### 2.5.3 Model Building and Validation

Using the final selected features and their LASSO coefficients, we further shrink these 88 features. LASSO compresses coefficients of unimportant features to zero, ultimately selecting the three most predictive features with nonzero coefficients for modeling. A linear prediction model (Rad‐score) is constructed. Subsequently, a support vector machine (SVM) is employed as the classifier, using Rad‐score as input for binary classification prediction of pCR status. Model performance was evaluated internally on the training set using 10‐fold cross‐validation, calculating the average area under the curve (AUC) and accuracy. The final model's generalization ability was tested on an independent validation set, plotting the receiver operating characteristic (ROC) curve and calculating AUC and accuracy.

### 2.6 Statistical Methods

Data analysis was performed using SPSS 23.0 and R 3.43 software (https://www.rproject.org/). Measurement data were tested for normality using the Kolmogorov–Smirnov method; data conforming to normal distribution were presented as mean ± standard deviation (*x* ± *s*) and compared using the independent samples *t*‐test (*χ* ^2^), while skewed distributions were expressed as median (upper and lower quartiles) and compared using the Mann–Whitney *U* test (*χ* ^2^). Qualitative data were expressed as *n* (%) and compared between groups using the *χ* ^2^ test or Fisher's exact test. Differences in patient characteristics between the training and test sets were analyzed. The diagnostic performance of radiomics scores was evaluated via ROC analysis, with statistical significance set at *p* < 0.05.

## 3 Results

### 3.1 Patient Characteristics of the pCR and npCR Groups

Among the 119 patients (mean age ± standard deviation: 49.5 ± 9.79 years), the sample was divided into a training set (*n* = 83; mean age ± standard deviation: 49.4 ± 10.30) and a test set (*n* = 36; mean age ± standard deviation: 49.6 ± 8.66). Histopathological analysis revealed that 43 patients (36.13%) achieved pCR, while 76 (63.87%) showed npCR. No statistically significant differences in clinical indicators were found between the training and test sets (*p* > 0.05), as presented in Tables 1 and 2.

**TABLE 1:** Comparison of the basic demographic and clinical data between the training and validation sets.

	Total		Training		Validation	
	npCR (*N* = 76)	pCR (*N* = 43)	npCR (*N* = 56)	pCR (*N* = 27)	npCR (*N* = 20)	pCR (*N* = 16)
Age (years)						
Mean (SD)	50.1 (10.1)	48.4 (9.25)	49.9 (10.3)	48.5 (10.4)	50.7 (9.64)	48.3 (7.33)
Median [Min, Max]	49.0 [27.0, 77.0]	49.0 [28.0, 66.0]	49.5 [27.0, 77.0]	49.0 [28.0, 66.0]	48.0 [32.0, 67.0]	50.5 [34.0, 59.0]
Menopause						
FALSE	38 (50.0%)	21 (48.8%)	28 (50.0%)	13 (48.1%)	10 (50.0%)	8 (50.0%)
TRUE	38 (50.0%)	22 (51.2%)	28 (50.0%)	14 (51.9%)	10 (50.0%)	8 (50.0%)
Family history						
False	65 (85.5%)	38 (88.4%)	50 (89.3%)	25 (92.6%)	15 (75.0%)	13 (81.3%)
True	11 (14.5%)	5 (11.6%)	6 (10.7%)	2 (7.4%)	5 (25.0%)	3 (18.8%)
Breast side						
Left	43 (56.6%)	25 (58.1%)	32 (57.1%)	16 (59.3%)	11 (55.0%)	9 (56.3%)
Right	33 (43.4%)	18 (41.9%)	24 (42.9%)	11 (40.7%)	9 (45.0%)	7 (43.8%)
ER						
Negative	26 (34.2%)	31 (72.1%)	19 (33.9%)	19 (70.4%)	7 (35.0%)	12 (75.0%)
Positive	50 (65.8%)	12 (27.9%)	37 (66.1%)	8 (29.6%)	13 (65.0%)	4 (25.0%)
PR						
Negative	30 (39.5%)	32 (74.4%)	22 (39.3%)	20 (74.1%)	8 (40.0%)	12 (75.0%)
Positive	46 (60.5%)	11 (25.6%)	34 (60.7%)	7 (25.9%)	12 (60.0%)	4 (25.0%)
Ki67						
Low_expression	7 (9.2%)	2 (4.7%)	5 (8.9%)	1 (3.7%)	2 (10.0%)	1 (6.3%)
Over_expression	69 (90.8%)	41 (95.3%)	51 (91.1%)	26 (96.3%)	18 (90.0%)	15 (93.8%)
Molecular_subtype						
Her2_overexpress	16 (21.1%)	7 (16.3%)	8 (14.3%)	5 (18.5%)	8 (40.0%)	2 (12.5%)
Luminal A	3 (3.9%)	3 (7.0%)	2 (3.6%)	2 (7.4%)	1 (5.0%)	1 (6.3%)
Luminal B	44 (57.9%)	19 (44.2%)	35 (62.5%)	10 (37.0%)	9 (45.0%)	9 (56.3%)
TN	13 (17.1%)	14 (32.6%)	11 (19.6%)	10 (37.0%)	2 (10.0%)	4 (25.0%)

**TABLE 2:** Statistical analyses of the training and validation sets.

	Total (*N* = 119)	Training (*N* = 83)	Validation (*N* = 36)	*p*
Age (years)				0.935
Mean (SD)	49.5 (9.79)	49.4 (10.3)	49.6 (8.66)	
Median [Min, Max]	49.0 [27.0, 77.0]	49.0 [27.0, 77.0]	48.5 [32.0, 67.0]	
Menopause				1
False	59 (49.6%)	41 (49.4%)	18 (50.0%)	
True	60 (50.4%)	42 (50.6%)	18 (50.0%)	
Family history				0.12
False	103 (86.6%)	75 (90.4%)	28 (77.8%)	
True	16 (13.4%)	8 (9.6%)	8 (22.2%)	
Breast_side				0.977
Left	68 (57.1%)	48 (57.8%)	20 (55.6%)	
Right	51 (42.9%)	35 (42.2%)	16 (44.4%)	
ER				0.616
Negative	57 (47.9%)	38 (45.8%)	19 (52.8%)	
Positive	62 (52.1%)	45 (54.2%)	17 (47.2%)	
PR				0.766
Negative	62 (52.1%)	42 (50.6%)	20 (55.6%)	
Positive	57 (47.9%)	41 (49.4%)	16 (44.4%)	
Ki67				1
Low_expression	9 (7.6%)	6 (7.2%)	3 (8.3%)	
Over_expression	110 (92.4%)	77 (92.8%)	33 (91.7%)	
Molecular_subtype				0.416
Her2_overexpress	23 (19.3%)	13 (15.7%)	10 (27.8%)	
Luminal A	6 (5.0%)	4 (4.8%)	2 (5.6%)	
Luminal B	63 (52.9%)	45 (54.2%)	18 (50.0%)	
TN	27 (22.7%)	21 (25.3%)	6 (16.7%)	
Label				0.301
npCR	76 (63.9%)	56 (67.5%)	20 (55.6%)	
pCR	43 (36.1%)	27 (32.5%)	16 (44.4%)	

### 3.2 Magnetic Resonance Imaging Histological Characterization

**FIGURE 1:**
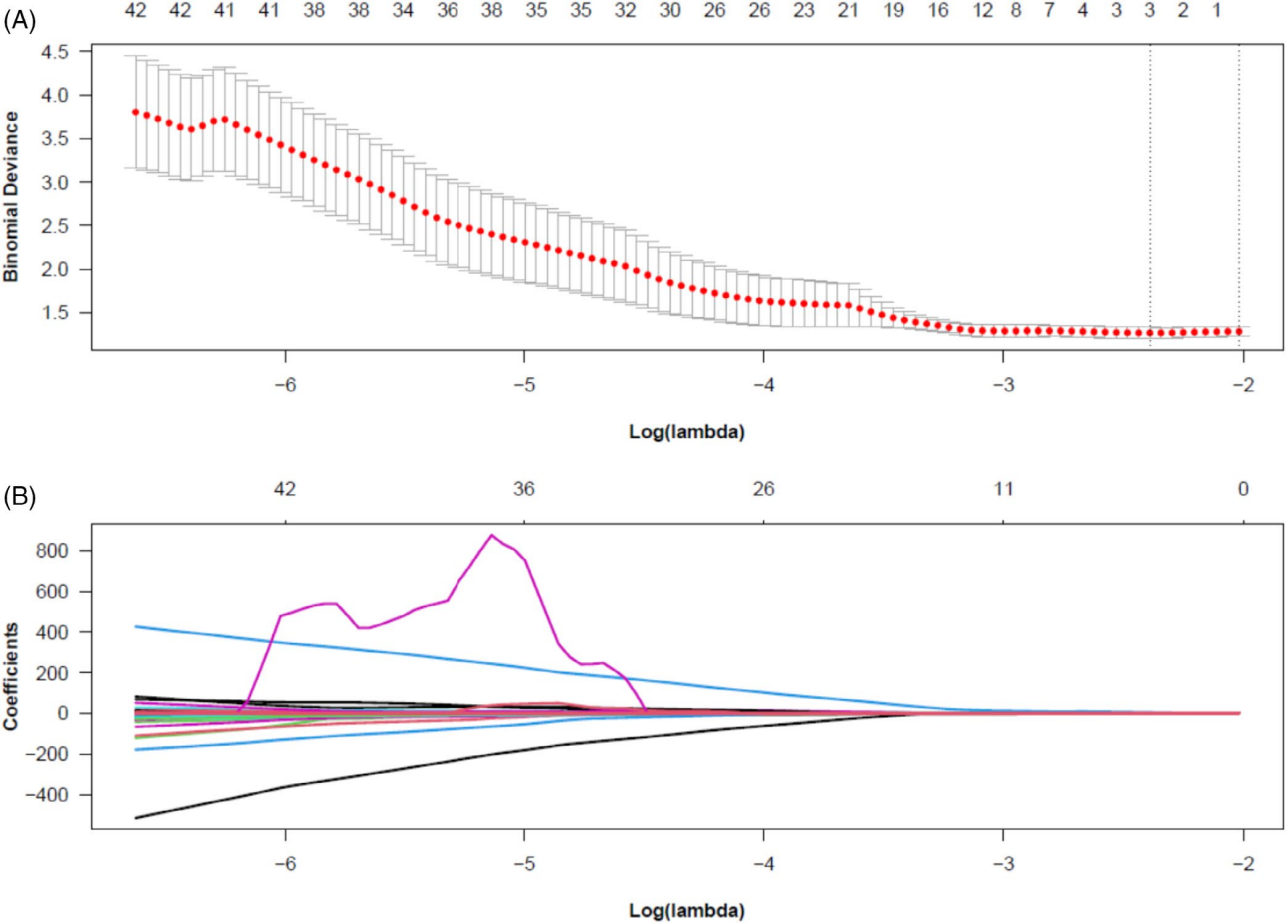
Feature selection process using the least absolute shrinkage and selection operator (LASSO) regression with 10‐fold cross‐validation. (A) Cross‐validation curve for tuning the LASSO penalty parameter (lambda). The *x*‐axis represents the logarithm of the lambda values (Log(*λ*)). The *y*‐axis represents the binomial deviance (a measure of model error) derived from 10‐fold cross‐validation; lower values indicate better model fit. The red dot indicates the position of the lambda value that gives the minimum mean cross‐validated error (*λ* min). The left vertical dashed line is drawn at Log(*λ* min). The right vertical dashed line is drawn at the value of Log(*λ*) where the deviance is within one standard error of the minimum (*λ* 1se), which selects a more parsimonious model. The numbers across the top of the plot indicate the number of nonzero coefficients (i.e., selected features) remaining in the model at each lambda value along the *x*‐axis. (B) LASSO coefficient shrinkage path. The *x*‐axis is the same Log(*λ*) scale as in (A). The *y*‐axis represents the standardized coefficient value for each feature. Each colored curve traces the change in the coefficient of one specific radiomics feature as the penalty (lambda) increases. As lambda increases (moving rightward), the LASSO algorithm shrinks the coefficients of less important features toward zero, effectively removing them from the model. The final three features with nonzero coefficients at the selected *λ* min (left dashed line) were chosen to build the radiomics signature. Their corresponding coefficient values are provided in the main text.

For each case, 851 initial radiomic features were extracted from DCE‐MRI images. After feature stability screening, intergroup difference analysis, and correlation‐based redundancy removal, 88 key features were retained for subsequent modeling. Ultimately, through LASSO regression (10‐fold cross‐validation), three optimal features with nonzero coefficients were selected from these 88 features (Figure 1, Table 3). These three features constitute the integral components of our radiomics signature and were used to construct the predictive model. The selected features and their corresponding LASSO coefficients were: wavelet. LLH_glcm_Idn (+4.2158), wavelet. LLH_glcm_MCC (+0.3262), and wavelet. LHL_firstorder_Skewness (−1.4202). The resulting LASSO regression equation was: *Y* = −5.1969542 + 4.2158041 × wavelet. LLH_glcm_Idn + 0.3262446 × wavelet. LLH_glcm_MCC − 1.4202265 × wavelet. LHL_firstorder_Skewness.

**TABLE 3:** Optimal imaging features screened by the LASSO algorithm.

Feature name	Characterization
Wavelet‐LLH_GLCM_Idn	Inverse difference normalization in grayscale covariance matrices based on wavelet transforms
Wavelet‐LLH_GLCM_MCC	Maximum correlation coefficient of the gray scale covariance matrix based on the wavelet transform
Wavelet‐LHL_firstorder_Skewness	Skewness of first‐order statistics based on wavelet transforms

### 3.3 Diagnostic Efficacy of the Model

**FIGURE 2:**
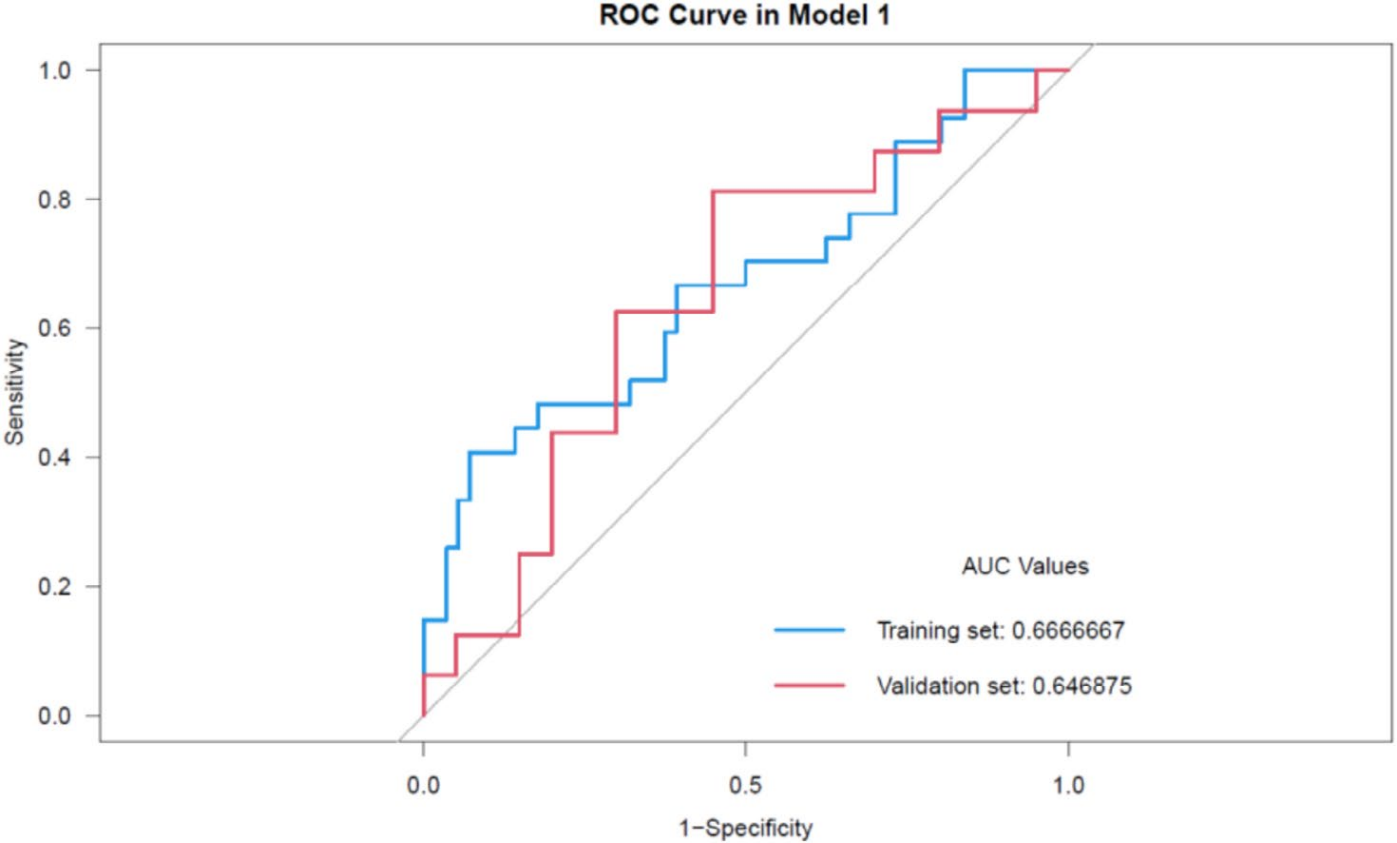
Receiver operating characteristics (ROC) curves and area under the curve (AUC) for the predictive model in both training and validation sets.

The ROC curves for models developed under varying regularization coefficients (*C*) were analyzed for both the training and test sets. The AUC changes were plotted separately (Figure 2), showing an AUC of 0.667 and an accuracy of 66.27% for the training set, while the validation set achieved an AUC of 0.647 and an accuracy of 73.49%.

### 3.4 Decision Curves

To evaluate the potential clinical utility of our radiomics model, we performed decision curve analysis (DCA), a statistical method that assesses the net benefit of using a prediction model to guide clinical decisions across a range of threshold probabilities. In this analysis, we considered a hypothetical clinical scenario in which the model might be used to identify patients with a high probability of pCR who could potentially be considered for de‐escalated surgical approaches in future clinical trials, although the current standard of care remains surgery for all patients.

**FIGURE 3:**
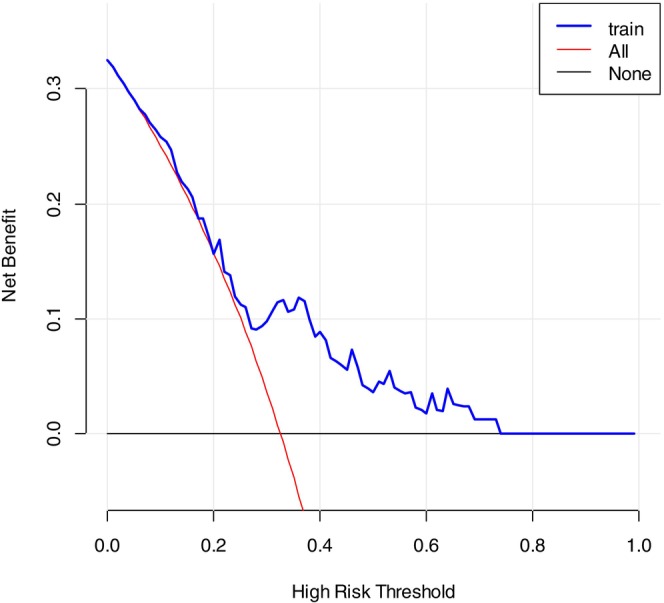
Decision curves for the training set, where the *x*‐axis represents threshold probability and the *y*‐axis reflects net benefit.

As shown in Figure 3, when the threshold probability for considering pCR was set above 0.3, the use of our model to guide decision‐making yielded a positive net benefit ranging from 3% to 8% compared to the default strategy of performing surgery on all patients. This indicates that, within this threshold range, the model could theoretically reduce unnecessary interventions without compromising patient outcomes. However, it is important to emphasize that this analysis is exploratory and based on retrospective data; the net benefit observed does not constitute evidence of clinical efficacy, and the model is not ready for clinical implementation. The DCA results should be interpreted as hypothesis‐generating and supportive of further prospective validation.

The model assumes that patients achieving pCR after neoadjuvant therapy may avoid surgery, whereas those not achieving pCR would require surgical intervention. The decision curve analysis (Figure 3) shows that, with a threshold probability > 0.3, the predictive model provides an incremental net benefit (ranging from 3% to 8%) over the strategy of performing surgery on all patients, with the net benefit increasing as the threshold probability rises.

## 4 Discussion

This study developed and validated a radiomics model for early prediction of pCR in breast cancer based on DCE‐MRI following the second cycle of NAC. The core finding is that a minimalist model comprising only three wavelet transform features achieved moderate yet stable predictive performance (AUC = 0.647) in an independent validation cohort. Its decision curve analysis suggests potential for clinical decision support at specific threshold values.

### 4.1 Key Findings and Clinical Implications

Achieving pCR is critical for improving breast cancer patient outcomes, and early, accurate prediction of pCR is essential for implementing personalized treatment [11, 12, 13]. This study focuses on imaging informatics analysis on the mid‐treatment phase (after the second cycle)—a key clinical decision point—aiming to explore a quantitative predictive method that precedes postoperative pathology and offers greater objectivity than traditional morphological imaging assessment. Our model confirms an association between mid‐treatment tumor radiomics features and final pCR status. Although its AUC value (0.647) is moderate and lower than some high‐performance literature reports combining multiple time points or clinical features (AUC often > 0.80) [14, 15, 16], its primary value lies in providing proof‐of‐concept for a simplified clinical scenario where prediction is achievable with a single mid‐treatment scan. Decision curve analysis indicated that, under hypothetical clinical scenarios and within a specific threshold probability range (0.3), the model could potentially offer net benefit compared to the “surgery for all” strategy. This finding suggests that the model may have potential for aiding clinical decision‐making, but this interpretation is strictly exploratory and requires confirmation in prospective studies. It must be clarified that this does not imply the model can currently be used to exempt patients from standard surgery [17]. Its significance lies in providing an objective, quantifiable screening tool for identifying patient subgroups with extremely high pCR probability who may be suitable for surgical de‐escalation exploration in future prospective clinical studies.

### 4.2 In‐Depth Analysis of Model Performance: Strengths, Limitations, and Attribution

We acknowledge that the absolute predictive performance of this study model requires improvement. A rational analysis of its performance is crucial, as it directly impacts the objective evaluation of its value and future directions for refinement. Factors influencing performance may be multifaceted:

First, study design and sample size: As a single‐center retrospective study, the limited sample size (*n* = 119) may restrict the model's ability to learn features from a broader population and poses a potential risk of overfitting. We employed rigorous feature selection (851 → 88 → 3 features) for dimensionality reduction, combined with LASSO regularization and an independent validation set. This resulted in highly consistent performance between the training and validation sets (AUC difference of 0.02), indicating good robustness of the internal validation. Empirical evidence suggests that overfitting was effectively controlled [18]; however, the single‐center design limits the model's generalizability. Future studies should incorporate multicenter data and assess the model's sensitivity to segmentation variations to comprehensively validate its robustness.

Second, feature strategy selection: This study intentionally adopted a single‐time‐point (mid‐treatment) analysis strategy, excluding pre‐ and posttreatment feature changes (delta‐radiomics). While this design simplifies clinical workflows and tests a clinically relevant question, it may sacrifice dynamic data containing important predictive information, potentially contributing to suboptimal performance [19, 20, 21]. Future models should integrate delta‐radiomics features to enhance performance. Moreover, validation across multiple time points—including baseline, mid‐treatment, and posttreatment scans—would be necessary to fully assess the model's predictive capability and to determine whether it truly complements conventional morphological assessment.

Third, the absence of comparative benchmarks: Due to retrospective data limitations, this study could not perform head‐to‐head comparisons with conventional MRI assessment parameters (e.g., RECIST criteria, ADC values). Literature indicates that predictive efficacy slightly falls below conventional preoperative MRI assessments (AUC ~0.707) [22]. This highlights a limitation of the present study while also suggesting that the advantage of radiomics may lie in its objective quantitative capabilities and ability to uncover tumor internal heterogeneity, rather than necessarily surpassing morphological measurements in this specific scenario. Future prospective studies must include such systematic comparisons.

Finally, regarding the biological basis of our model: The three selected features are not merely statistical constructs but are integral components that provide insights into tumor biology. All three are texture features derived from wavelet transforms, which capture image details across multiple spatial scales—a capability that conventional visual assessment lacks.

wavelet. LLH_glcm_Idn (Inverse Difference Normalized) measures local image homogeneity. Higher values indicate greater uniformity in the local gray‐level distribution, which may reflect reduced cellular density, increased fibrosis, or more homogeneous microvascular permeability after chemotherapy. Its positive coefficient suggests that tumors achieving pCR tend to develop more locally homogeneous texture patterns after two cycles of NAC.

wavelet. LLH_glcm_MCC (Maximum Correlation Coefficient) quantifies the complexity and predictability of texture patterns. Higher MCC values indicate more complex, less predictable patterns, which may correspond to heterogeneous treatment effects within the tumor—such as mixed areas of necrosis, fibrosis, and residual viable tumor. Its positive coefficient suggests that pCR tumors exhibit greater overall textural complexity.

wavelet. LHL_firstorder_Skewness measures the asymmetry of the voxel intensity distribution. Negative skewness (a left‐tailed distribution) indicates a shift toward higher signal intensity values, which on DCE‐MRI may reflect increased contrast uptake in responding tumors due to altered vascular permeability or reduced cellular density allowing greater contrast distribution. Its negative coefficient suggests that pCR tumors tend to have a greater proportion of high‐signal voxels.

Collectively, these features suggest that pCR is associated with a specific pattern of textural evolution during early treatment: increased local homogeneity alongside increased global complexity, with a shift toward higher signal intensity. These imaging phenotypes likely reflect underlying biological processes, including tumor cell kill, stromal remodeling, and vascular changes. Previous studies have similarly reported that posttreatment texture heterogeneity correlates with chemotherapy sensitivity [23, 24], which is consistent with our findings.

Although the validation set accuracy reached 73.49%, this still implies that the pCR status of more than one‐quarter of patients was misclassified, highlighting that the model is not yet sufficient to independently guide clinical decision‐making at this stage. Further improvements in accuracy will require a larger sample size, multicenter data, and feature integration strategies.

### 4.3 Limitations of the Study and Future Prospects

This study has several limitations that should be fully considered when interpreting the results. First, the absence of reproducibility assessment represents a significant methodological limitation. Although segmentation was performed by experienced radiologists and cross‐checked, there was no quantitative evaluation of intra‐ and inter‐observer consistency (e.g., ICC, Dice coefficient). Radiomic features are sensitive to ROI delineation, and variability in this step is a key factor affecting model generalization. Second, clinical information integration was insufficient. Aiming to explore the potential of pure imaging features, this study excluded strong prognostic factors such as molecular subtypes and Ki‐67 from the model. Developing a multimodal fusion model integrating imaging, clinical, and pathological data represents an essential direction for enhancing predictive efficacy and clinical applicability [25, 26]. Third, external validity remains unclear. Furthermore, the relatively small sample size of the validation set (*n* = 36) limits the precision of the performance evaluation; based on the accuracy estimate from the validation set, the false positive rate is approximately 26.5%. Such a high false positive rate implies that if the model is used to identify patients eligible for surgical exemption, it may result in some patients who have not achieved pCR losing the opportunity for curative treatment. Future work should aim to reduce the false positive rate through more advanced feature selection and model calibration. Fourth, the lack of multi‐time‐point validation limits our ability to claim that the model complements morphological assessment. While our single‐time‐point analysis demonstrates feasibility, future studies should include longitudinal data to enable delta‐radiomics analysis and to compare model performance with conventional imaging metrics across multiple time points. Fifth, The decision curve analysis presented in this study is a statistical exploration of potential net benefit under hypothetical scenarios and should not be interpreted as evidence of clinical efficacy. Prospective studies with appropriate clinical trial designs are necessary to determine whether use of this model would actually improve patient outcomes.

Based on the above discussion, future research should follow a clear path: The primary task is to conduct multicenter, prospective external validation to assess the model's robustness and generalizability. Second, efforts should focus on developing integrated models that combine pre‐ and posttreatment delta‐radiomics features, key clinical‐pathological variables, and promising conventional imaging metrics. Such multi‐time‐point and multi‐modal approaches will be essential to determine whether radiomics truly adds value to morphological assessment at a single time point. Finally, standardization of imaging acquisition, segmentation, and feature extraction workflows must be advanced, as this constitutes the cornerstone for radiomics to transition into clinical application [27, 28].

In summary, this study confirms the feasibility and potential clinical value of radiomics features derived from mid‐treatment MRI in predicting pCR following NAC for breast cancer. The three wavelet‐based texture features identified in this study—wavelet. LLH_glcm_Idn, wavelet. LLH_glcm_MCC, and wavelet. LHL_firstorder_Skewness—are integral components of our radiomics signature and provide a quantitative window into treatment‐induced changes in tumor heterogeneity that are not accessible through conventional morphological assessment. Although this preliminary, single‐center exploratory model demonstrates moderate performance and faces several limitations, it offers new insights and objective evidence for achieving earlier personalized treatment response assessment. The significance of this study lies not only in the predictive model it generated but also in identifying the key issues and validation steps that must be addressed to advance toward greater clinical utility in this field.

## Author Contributions

**Hongen Li:** writing – original draft. **Li Zhang:** software.**Yihui Zeng:** data curation, investigation. **Yuanyuan Chen:** investigation. **Xiaosong Jiang:** validation. **Ruoxian Zhang:** visualization. **Yan Zhang:** writing – review and editing.

## Funding

This study was supported by the Fund of Guangzhou Science and Technology Plan Project (grant no. 202002030217).

## Ethics Statement

This study was approved by the Ethics Review Committee of Guangdong Women and Children Hospital (Approval No. [202401244]). Due to the retrospective nature of the study and the use of anonymized data, the requirement for informed patient consent was waived. This waiver complies with the Declaration of Helsinki and relevant Chinese ethical guidelines for retrospective research.

## Consent

The authors have nothing to report.

## Conflicts of Interest

The authors declare no conflicts of interest.

## Data Availability

The data that support the findings of this study are available from the corresponding author upon reasonable request.

## References

[1] R. S. Patwardhan , A. Rai , D. Sharma , S. K. Sandur , and S. Patwardhan , “Txnrd1 as a Prognosticator for Recurrence, Metastasis and Response to Neoadjuvant Chemotherapy and Radiotherapy in Breast Cancer Patients,” Heliyon 10, no. 6 (2024): e27011. 38524569 10.1016/j.heliyon.2024.e27011 PMC10958228

[2] J. Q. Freeman , S. Shubeck , F. M. Howard , N. Chen , R. Nanda , and D. Huo , “Evaluation of Multigene Assays as Predictors for Response to Neoadjuvant Chemotherapy in Early‐Stage Breast Cancer Patients,” npj Breast Cancer 9, no. 1 (2023): 33. 37149628 10.1038/s41523-023-00536-z PMC10164191

[3] L. Kovacevic , M. Petrovecki , L. Korsa , Z. Marusic , I. Dumic‐Cule , and M. Prutki , “Early Assessment of Neoadjuvant Chemotherapy Response Using Multiparametric Magnetic Resonance Imaging in Luminal B‐Like Subtype of Breast Cancer Patients: A Single‐Center Prospective Study,” Diagnostics 13, no. 4 (2023): 694. 36832182 10.3390/diagnostics13040694 PMC9955433

[4] Y. J. Qi , G. H. Su , C. You , et al., “Radiomics in Breast Cancer: Current Advances and Future Directions,” Cell Reports Medicine 5, no. 9 (2024): 101719. 39293402 10.1016/j.xcrm.2024.101719 PMC11528234

[5] M. Wang , W. Chen , R. Ren , Y. Lin , J. Tang , and M. Wu , “Comparative Analysis of Multi‐Zone Peritumoral Radiomics in Breast Cancer for Predicting NAC Response Using ABVS‐Based Deep Learning Models,” Frontiers in Oncology 15 (2025): 1586715. 40438687 10.3389/fonc.2025.1586715 PMC12116539

[6] J. Zhang , Q. Wu , P. Lei , X. Zhu , and B. Li , “MRI‐Based Radiomics Models for Early Predicting Pathological Response to Neoadjuvant Chemotherapy in Triple‐Negative Breast Cancer: A Systematic Review and Meta‐Analysis,” Journal of Applied Clinical Medical Physics 26, no. 10 (2025): e70296. 41094242 10.1002/acm2.70296 PMC12527642

[7] Z. Wu , Q. Lin , G. Fu , et al., “Pretreatment MRI‐Based Radiomics for Predicting Recurrence and Disease‐Free Survival in Young Women With Breast Cancer After Neoadjuvant Chemotherapy,” Journal of Computer Assisted Tomography 50, no. 3 (2026): 390–402. 41305861 10.1097/RCT.0000000000001827

[8] A. E. Giuliano , S. B. Edge , and G. N. Hortobagyi , “Eighth Edition of the AJCC Cancer Staging Manual: Breast Cancer,” Annals of Surgical Oncology 25, no. 7 (2018): 1783–1785. 29671136 10.1245/s10434-018-6486-6

[9] K. N. Ogston , I. D. Miller , S. Payne , et al., “A New Histological Grading System to Assess Response of Breast Cancers to Primary Chemotherapy: Prognostic Significance and Survival,” Breast 12, no. 5 (2003): 320–327. 14659147 10.1016/s0960-9776(03)00106-1

[10] C. Shintia , H. Endang , and K. Diani , “Assessment of Pathological Response to Neoadjuvant Chemotherapy in Locally Advanced Breast Cancer Using the Miller‐Payne System and TUNEL,” Malaysian Journal of Pathology 38, no. 1 (2016): 25–32. 27126661

[11] A. Stenmark Tullberg , S. Woxlin , F. Sjölin , et al., “Predicting Immune Responsiveness in ER‐Positive Breast Cancer for Personalized Therapy: A Population‐Based Study,” npj Precision Oncology 9, no. 1 (2025): 250. 40702082 10.1038/s41698-025-01035-z PMC12287262

[12] M. Zafrakas , I. Gavalas , P. Papasozomenou , C. Emmanouilides , and M. Chatzidimitriou , “Proteomics in Diagnostic Evaluation and Treatment of Breast Cancer: A Scoping Review,” Journal of Personalized Medicine 15, no. 5 (2025): 177. 40423049 10.3390/jpm15050177 PMC12113354

[13] C. Tinterri , B. Fernandes , A. Zambelli , et al., “The Impact of Different Patterns of Residual Disease on Long‐Term Oncological Outcomes in Breast Cancer Patients Treated With Neo‐Adjuvant Chemotherapy,” Cancers 16, no. 2 (2024): 376. 38254865 10.3390/cancers16020376 PMC10814808

[14] S. Xu , Y. Ying , Q. Hu , et al., “Fusion Model Integrating Multi‐Sequence MRI Radiomics and Habitat Imaging for Predicting Pathological Complete Response in Breast Cancer Treated With Neoadjuvant Therapy,” Cancer Imaging 25, no. 1 (2025): 108. 40883826 10.1186/s40644-025-00929-2 PMC12395702

[15] Y. Yu , Z. Wang , Q. Wang , et al., “Radiomic Model Based on Magnetic Resonance Imaging for Predicting Pathological Complete Response After Neoadjuvant Chemotherapy in Breast Cancer Patients,” Frontiers in Oncology 13 (2024): 1249339. 38357424 10.3389/fonc.2023.1249339 PMC10865896

[16] Q. X. Cui , L. Q. Zhou , X. Y. Wang , et al., “Novel MRI‐Based Hyper‐Fused Radiomics for Predicting Pathologic Complete Response to Neoadjuvant Therapy in Breast Cancer,” Academic Radiology 32, no. 5 (2025): 2477–2488. 39765433 10.1016/j.acra.2024.12.043

[17] X. Liu , F. Xu , K. Zhao , et al., “Comprehending the Cuproptosis and Cancer‐Immunity Cycle Network: Delving Into the Immune Landscape and Its Predictive Role in Breast Cancer Immunotherapy Responses and Clinical Endpoints,” Frontiers in Immunology 15 (2024): 1344023. 38312844 10.3389/fimmu.2024.1344023 PMC10834629

[18] C. Xu , P. Coen‐Pirani , and X. Jiang , “Empirical Study of Overfitting in Deep Learning for Predicting Breast Cancer Metastasis,” Cancers 15, no. 7 (2023): 1969. 37046630 10.3390/cancers15071969 PMC10093528

[19] X. Li , C. Li , H. Wang , L. Jiang , and M. Chen , “Comparison of Radiomics‐Based Machine‐Learning Classifiers for the Pretreatment Prediction of Pathologic Complete Response to Neoadjuvant Therapy in Breast Cancer,” PeerJ 12 (2024): e17683. 39026540 10.7717/peerj.17683 PMC11257043

[20] Y. H. Huang , Z. Y. Shi , T. Zhu , et al., “Longitudinal MRI‐Driven Multi‐Modality Approach for Predicting Pathological Complete Response and B Cell Infiltration in Breast Cancer,” Advanced Science 12, no. 12 (2025): e2413702. 39921294 10.1002/advs.202413702 PMC11948082

[21] J. Zhang , Q. Wu , W. Yin , et al., “Development and Validation of a Radiopathomic Model for Predicting Pathologic Complete Response to Neoadjuvant Chemotherapy in Breast Cancer Patients,” BMC Cancer 23, no. 1 (2023): 431. 37173635 10.1186/s12885-023-10817-2 PMC10176880

[22] M. Caballo , W. B. G. Sanderink , L. Han , Y. Gao , A. Athanasiou , and R. M. Mann , “Four‐Dimensional Machine Learning Radiomics for the Pretreatment Assessment of Breast Cancer Pathologic Complete Response to Neoadjuvant Chemotherapy in Dynamic Contrast‐Enhanced MRI,” Journal of Magnetic Resonance Imaging 57, no. 1 (2023): 97–110. 35633290 10.1002/jmri.28273 PMC10083908

[23] Y. Li , F. Yang , Z. Pan , et al., “VARIDT 4.0: Distribution Variability of Drug Transporters,” Nucleic Acids Research 54, no. D1 (2026): D1702–D1710. 41036573 10.1093/nar/gkaf981 PMC12807592

[24] J. R. Teruel , M. G. Heldahl , P. E. Goa , et al., “Dynamic Contrast‐Enhanced MRI Texture Analysis for Pretreatment Prediction of Clinical and Pathological Response to Neoadjuvant Chemotherapy in Patients With Locally Advanced Breast Cancer,” NMR in Biomedicine 27, no. 8 (2014): 887–896. 24840393 10.1002/nbm.3132

[25] Z. Wu , J. Y. Yang , C. B. Yan , C. G. Zhang , and H. C. Yang , “Integrating SAM Priors With U‐Net for Enhanced Multiclass Cell Detection in Digital Pathology,” Scientific Reports 15, no. 1 (2025): 15641. 40325120 10.1038/s41598-025-99278-0 PMC12052837

[26] Y. Zhou , F. Jin , G. Suo , and J. Yang , “ResViT‐GANNet: A Deep Learning Framework for Classifying Breast Cancer Histopathology Images Using Multimodal Attention and GAN‐Based Augmentation,” BMC Medical Imaging 25, no. 1 (2025): 401. 41023673 10.1186/s12880-025-01940-6 PMC12482568

[27] L. Garrucho , K. Kushibar , C. A. Reidel , et al., “A Large‐Scale Multicenter Breast Cancer DCE‐MRI Benchmark Dataset With Expert Segmentations,” Scientific Data 12, no. 1 (2025): 453. 40108146 10.1038/s41597-025-04707-4 PMC11923173

[28] Y. Yu , G. F. Li , W. X. Tan , et al., “Towards Automatical Tumor Segmentation in Radiomics: A Comparative Analysis of Various Methods and Radiologists for Both Region Extraction and Downstream Diagnosis,” BMC Medical Imaging 25, no. 1 (2025): 63. 40000987 10.1186/s12880-025-01596-2 PMC11863488

